# Perioperative Mortality Risk in Patients Undergoing Transoral Robotic Surgery for T1-T2 Oropharyngeal Squamous Cell Carcinoma: A National Cancer Database Study

**DOI:** 10.3389/fonc.2021.808465

**Published:** 2022-01-06

**Authors:** Joel C. Davies, Zain Husain, Terry A. Day, Evan M. Graboyes, Antoine Eskander

**Affiliations:** ^1^ Department of Otolaryngology-Head and Neck Surgery, Sinai Health System, University of Toronto, Toronto, ON, Canada; ^2^ Department of Otolaryngology-Head & Neck Surgery, Sunnybrook Health Science Center, Odette Cancer Center, University of Toronto, Toronto, ON, Canada; ^3^ Department of Otolaryngology-Head & Neck Surgery, Medical University of South Carolina, Charleston, SC, United States; ^4^ Department of Public Health Sciences, Medical University of South Carolina, Charleston, SC, United States

**Keywords:** transoral robotic surgery (TORS), oropharyngeal cancer, mortality, morbidity, head and neck cancer (HN)

## Abstract

The National Cancer Database is a joint project of the Commission on Cancer of the American College of Surgeons and the American Cancer Society. The American College of Surgeons and the Commission on Cancer have not verified and are not responsible for the analytic or statistical methods used or for the conclusions drawn from these data by the investigators.

**Introduction:**

Transoral robotic surgery (TORS) is well established as initial definitive treatment for early-stage oropharyngeal squamous cell carcinoma (OPSCC) as an alternative to radiation therapy with similar survival rates. While proponents of TORS focus on the reduced morbidity of treatment compared to open procedures, shortened hospital admissions and equivalent survival outcomes to non-surgical treatment, there remain concerns over the risk of mortality within the acute perioperative period. Therefore, we sought to determine the 30-day and 90-day perioperative mortality risk using the National Cancer Database.

**Methods:**

A retrospective cohort analysis was performed for patients diagnosed with pathologic T1/2 OPSCC between January 1, 2010, and December 31, 2016 that underwent primary surgical treatment with TORS and was not restricted by HPV status. The primary outcome was 30-day perioperative mortality. The secondary outcome was 90-day perioperative mortality. Univariable analysis was used to identify variables associated with 30-day perioperative mortality.

**Results:**

In total, 4,127 patients (mean [SD; range] age, 59 [9.5; 22-90] years; 3,476 [84%] men and 651 [16%] women) met inclusion criteria. The number of patients with pT1-2 OPSCC undergoing TORS increased three-fold between 2010 (279/4,127; 7%) to 2016 (852/4,127; 21%). The overall 30-day and 90-day perioperative mortality rate for TORS during the study period was 0.6% (23/4,127) and 0.9% (38/4,127), respectively. On univariable analysis (UVA), age≥65 was the only predictor of 30-day perioperative mortality (OR 3.41; 95% CI 1.49-7.81).

**Conclusion:**

The overall risk of all cause mortality following TORS for early-stage OPSCC remains low. The risk of mortality is higher in elderly patients and should be considered, in addition to previously established risk factors, during patient selection and counselling.

## Introduction

Early stage oropharyngeal carcinoma (OPSCC) has an overall good prognosis for cure with either definitive radiation therapy (RT) or definitive surgery. Human Papillomavirus (HPV) positive OPSCC numbers continue to rise leading to an increasing utilization of transoral robotic surgery (TORS) for surgical treatment in place of transoral laser microsurgery (TLM) and/or open surgical resection. Within the last two decades, a paradigm shift has occurred in the management of T1-T2 OPSCC. TORS has permitted minimal access surgical treatment of oropharyngeal tumors without the added morbidity of traditional open approaches (1) and, in selected low-risk HPV positive patients, can obviate the need for adjuvant treatment (2). As such, from 2004 to 2013, national level data from the USA demonstrates that there was a 26% increase in the use of primary surgery for T1/2 OPSCC (3). This dramatic increase has been attributed to patient preference for TORS as it has been touted as an alternative that is safe, effective and requiring brief hospital admissions. More recently, however, trends in use of surgery, in the HPV+ setting, declined from 2010 to 2014 due to more stringent patient selection with a decrease in triple modality exposure for those treated with primary surgery (4). Despite these advances, the majority of patients receive primary concurrent chemoradiotherapy (4). This suggests that the head and neck oncology community is still assessing the role and place for TORS in the management of patients with T1-T2 OPSCC.

Although survival outcomes are equivalent between primary chemoradiation and TORS for T1-T2 HPV+ oropharynx cancer (2, 5), one ongoing concern for TORS has been the potential added risk of morbidity and mortality. Life threatening hemorrhage and airway compromise in the immediate and subsequent postoperative period remain the most feared complications of TORS. Estimates of hemorrhage from all sources range from 7-22% (6, 7). Since the introduction of TORS as a treatment modality, institutional protocols and a variety of surgical strategies have been developed to reduce the likelihood of life threatening complications (8–10). For example, one recent randomized controlled surgical trial modified study protocols during the study period to strongly recommend that TORS patients undergo prophylactic tracheotomy for airway protection (10). Likewise, many protocols rely on transcervical arterial ligation (TAL) of various branches of the external carotid during the neck dissection to decrease the likelihood of life threatening hemorrhage (7, 8, 11).

The reported incidence of perioperative mortality for patients with head and neck cancer undergoing TORS ranges from 0.07-3.3% (**Table 1**) (2, 7, 14, 16–19). Although these studies provide a general estimate, no study has specifically examined perioperative mortality as a primary outcome of TORS using national or hospital-based data. Therefore, the NCDB is the largest national database that can be used to provide an accurate estimate of perioperative mortality. The primary objective of this study was to determine 30-day perioperative mortality following TORS for early-stage OPSCC using data from the NCDB. Secondarily, we calculated the 90-day perioperative mortality.

**Table 1:** Overview of literature examining mortality in patients undergoing TORS.

Author (Year), Country	Study Design	Inclusion Criteria	Exclusion Criteria	Sample Size	Mortality no. (%)
de Almeida (12), USA	Retrospective review	1. SCC of the posterior oral cavity, oropharynx, larynx and hypopharynx 2. January 1, 2007-December 31, 2012	Not identified	410	1/410 (0.2%)^†^
Aubry et al. (13), France	Multi-Institutional Retrospective	1. All head and neck cancer sites, stages, tumor location treated with TORS 2. March 2009-December 2014	Not identified	178	2/178 (1.1%)^†^
Nichols et al. (10), Canada	Multi-Institutional Prospective Randomized Study (ORATOR1)	1. OPSCC, cT1-2/N0-2/M0 2. Aug 10, 2012-June 9, 2017	1. Medical comorbidities precluding radiotherapy, chemotherapy or surgery 2. Prior head and neck cancer 3. Prior head and neck radiation 4. Distant metastatic disease	68	1/68 (1.5%)^†^
Stokes et al. (7), USA	Meta-Analysis and Systematic Review	1. OPSCC (tonsil) 2. January 1, 2009-March 30, 2019	1. Case reports or case series (N<10) 2. Animal/cadaveric studies	1494	1/1494 (0.07%)^†^
Nguyen et al. (1), USA	Retrospective NCDB Study	1. OPSCC, cT1/2/N0-3/M0 2. January 1, 2010-December 31, 2015	1. Unknown surgical approach 2. Surgery not performed at diagnosing facility 3. Radiation or chemotherapy prior to TORS	2,658	38/2,658 (1.4%)*
Palma et al. (14), Canada	Multi-Institutional Prospective Randomized Study (ORATOR2)	1. HPV+ OPSCC, cT1-2/N0-2 2. February 2018-November 2020	1. Prior head and neck cancer 2. Prior head and neck radiation 3. Distant metastatic disease	61	2/61 (3.3%)^†^
Ferris et al. (15), USA	Multi-Institutional Prospective Randomized Study (ECOG3311)	3. HPV+ OPSCC, cT1-2 4. August 2013-September 2020	1. HPV- OPSCC 2. Matted nodes 3. Contraindication to TORS	359	1/359 (0.3%)^†^

*Based on 90-day perioperative mortality.

^†^Mortality rate for entire study period.

## Methods

### Data Source

Deidentified patient data from the National Cancer Database (NCDB) were used for this study. The NCDB is a hospital-based cancer registry that is a joint program of the American College of Surgeons Commission on Cancer and the American Cancer Society. The NCDB collects data from more than 1500 Commission on Cancer–accredited hospitals in the USA and include more than 70% of newly diagnosed cancer cases in the USA (20). Although the NCDB is not a population-based database, such as the Surveillance, Epidemiology, and End Results database, it reflects characteristics of population-based data in terms of demographic characteristics, staging, and treatment for patients with HNSCC (21). Given that this study used a public de-identified database, this study was exempt from review by the institutional review board at the Medical University of South Carolina. No one received compensation or was offered any incentive for participating in this study.

### Cohort Selection

All patients 18 years or older undergoing primary surgery using a robotic approach and neck dissection for pT1/2 N0-3 M0 oropharynx cancer (International Classification of Diseases for Oncology, Third Edition [ICD-O-3] codes C01.9, C02.1, C02.2, C02.4, C02.8, C02.9, C05.1, C09.0, C09.1, C09.8, C09.9, C10.0, C10.1, C10.2, C10.3, C10.8, C10.9, C14.0 and C14.2) of squamous cell carcinoma histologies (ICD-O-3 codes 8050-52, 8070-8074 and 8083) diagnosed between January 1, 2010, and December 31, 2016, were included. All HPV statuses (positive, negative or unknown) were included. Using the variable “Approach – Surgery of the Primary Site”, we selected only those patients that had undergone “robotic assisted” surgery. These patients were classified as having undergone TORS. Patients who were treated with open surgery, TORS converted to open surgery, an exclusively diagnostic procedure or unknown surgical approach were excluded. We also excluded those patients that had received radiation, chemotherapy or combined chemoradiation prior to surgery. Lastly, those patients with missing data regarding mortality or vital status were excluded.

### Statistical Analysis

Clinical, pathologic and treatment data were characterized with summary statistics (e.g. proportion for categorical variables and mean and standard deviation for continuous variables). The primary outcome was 30-day perioperative mortality. The secondary outcome was 90-day perioperative mortality. The 90-day mortality was calculated to ensure that delayed mortality events secondary to surgery were captured. Ordinary least-squares regression was used to estimate 30-day and 90-day mortality. Univariable analysis was used to identify variables which are associated to 30-day mortality. Multivariable regression was not performed given a low event rate. Sensitivity analysis was performed to assess impact of clinical staging on perioperative mortality compared to pathologic staging. Statistical analyses were performed using SPSS, version 25.0 (Armonk, NY: IBM Corp). Statistical significance was set at *P* < .05.

## Results

### Patient Cohort

In total, 4,127 patients (mean [SD; range] age, 59 [9.5; 22-90] years; 3,476 [84%] men and 651 [16%] women) meeting inclusion criteria underwent TORS from 2010-2016 for pT1/2 OPSCC. Demographic and clinicopathologic data including details regarding adjuvant treatment are summarized in **Table 2**. The number of patients with pT1-2 OPC undergoing TORS increased three-fold between 2010 (279/4,127; 7%) to 2016 (852/4,127; 21%). Most patients underwent TORS for resection of a tonsil primary (56%; 2,328/4,127), with the remainder divided between base of tongue (39%; 1,591/4,127) and any other subsite of the oropharynx (5%; 208/4,127).

**Table 2:** Baseline characteristics of pT1-2 OPSCC treated with TORS.

Characteristic	Overall, No. (%)
Age, mean (SD), y	59.3 (9.5)
<65	2982 (72)
≥65	1145 (28)
Sex (Male)	3476 (84)
Race	
White	3833 (93)
Black	187 (5)
Unknown	107 (2)
Charlson-Deyo comorbidities	
0	3282 (79)
1	644 (16)
2	136 (3)
≥3	65 (2)
Anatomic subsite	
BOT	1591 (39)
Tonsil	2328 (56)
Other	208 (5)
Facility type	
Academic/research program	3455 (84)
Comprehensive community cancer program	374 (9)
Community cancer program or integrated network	237 (7)
Pathologic T category*	
T1	2137 (52)
T2	1990 (48)
Pathologic N category*	
N0	843 (20)
N1	721 (18)
N2a	857 (21)
N2b	1476 (36)
N2c	112 (2)
N3	118 (3)
HPV status	
Positive	2769 (67)
Negative	524 (13)
Unknown	834 (20)
Margin status	
Positive	510 (13)
Negative	3520 (87)
Lymphovascular invasion	
No	3203 (78)
Yes	924 (22)
Extranodal extension	
No	2954 (72)
Yes	1173 (28)
Treatment modality	
Surgery	1465 (35)
Surgery + RT	2662 (45)
Surgery + CRT	817 (20)

*Staging based on AJCC 6 and 7.

Pathologic evaluation demonstrated that negative margins were achieved in 87% (3,538/4,127) with microscopic residual tumor in 8% (306/4,127), macroscopic tumor in 0.4% (16/4,127) and the degree of involvement was not specified in 5% (193/4,053). Lymphovascular invasion was identified in 22% (924/4,127) and unknown/indeterminate in 13% (551/4,127). Pathologic staging demonstrated pT1 in 52% (2,137/4,127) and pT2 in 48% (1,990/4,127). Nodal metastases were present in 80% (3,284/4,127) of patients. The majority (36%; 1,476/4127) of patients had multiple ipsilateral involved nodes. Extranodal extension was identified in 28% (1,173/4,127) of patients.

### Perioperative Mortality

**Figure 1:**
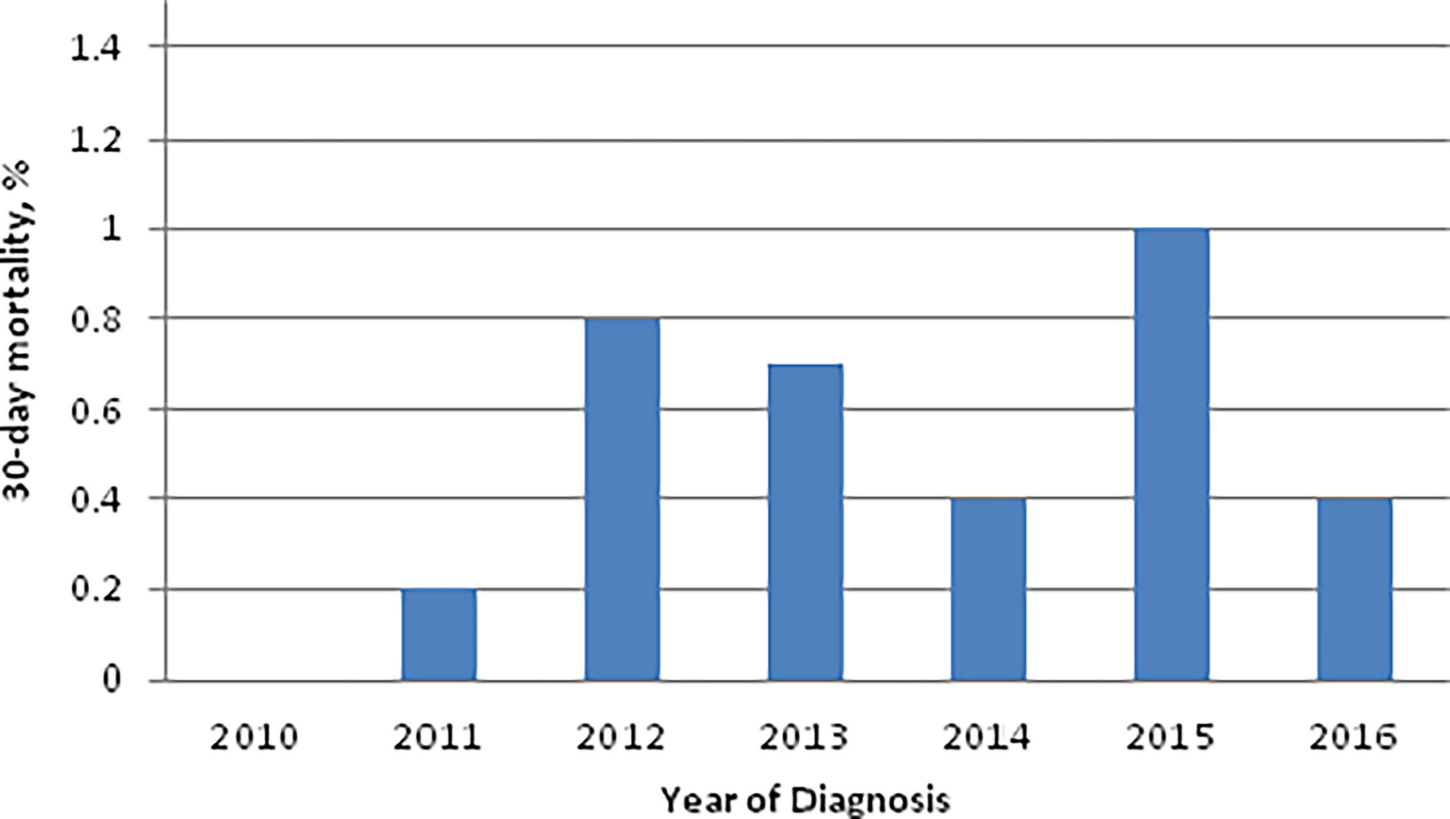
30-day mortality (%) for patients undergoing TORS for pT1/2 OPSCC distributed by year.

The overall 30-day and 90-day perioperative mortality rate for TORS during the study period was 0.6% (23/4,127) and 0.9% (38/4,127), respectively. The 30-day perioperative mortality rates for TORS during the study period were ≤1% for all years in the study period (**Figure 1**). The 30-day perioperative mortality rate did not differ based on anatomic subsite [Tonsil 0.6% (14/2328); base of tongue 0.5% (8/1591); other subsite 0.5% (1/208)] or pathologic T-category [pT1 0.6% (12/2125); pT2 0.6% (11/1979)]. On univariable analysis (UVA), age greater than 65 was the only predictor of 30-day perioperative mortality (OR 3.41; 95% CI 1.49-7.81; **Table 3**).

**Table 3:** Univariable analysis of 30-day perioperative mortality for pT1-2 OPSCC treated with TORS.

Characteristic	OR (95% CI)	P value
Age		
<65	1.00 [Reference]	
≥65	3.41 (1.49-7.81)	.002
Sex		
Male	1.00 [Reference]	N/A
Female	0.80 (0.24-2.70)	.72
Race		
White	1.00 [Reference]	N/A
Black	3.11 (0.95-10.55)	0.06
Unknown	0.97 (0.97-0.98)	0.56
Charlson-Deyo comorbidities		
0	1.00 [Reference]	N/A
1	2.94 (1.23-7.03)	.01
≥2	1.17 (0.15-8.92)	.88
Anatomic subsite		
BOT	1.00 [Reference]	N/A
Tonsil	1.20 (0.5-2.86)	.69
Other	1.05 (0.13-8.41)	.97
Facility type		
Academic/research program	1.00 [Reference]	N/A
Comprehensive community cancer program	1.46 (0.43-4.97)	.54
Community cancer program or integrated network	0.77 (0.10-5.75)	.79
Pathologic T category		
T1	1.00 [Reference]	N/A
T2	0.98 (0.43-2.24)	.97
Pathologic N category		
N0/1	1.00 [Reference]	N/A
N2/3	0.39 (0.17-0.90)	.02
HPV Status		
Negative	1.00 [Reference]	N/A
Positive	0.47 (0.15-1.51)	.19
Margin Status		
Negative	1.00 [Reference]	N/A
Positive	1.92 (0.71-5.19)	.19

NA, Not Applicable.

### Sensitivity Analysis

A sensitivity analysis was performed to assess the impact of clinical staging on perioperative mortality in comparison to pathologic staging. A total of 187 patients were down-staged to pT1/2 from initial clinical stage of cT3 (73/4,127) and cT4 (14/4,127). Although pathologic T3/4 patients were excluded, we performed a separate analysis of all TORS patients to evaluate those that were clinically staged T1/2, but up-staged based on pathology. This analysis demonstrated that 168 patients were initially staged as cT1/2, but were up-staged to pT3 (167/168) and pT4 (1/168). Within this group, the 30-day and 90-day perioperative mortality rate was 0.6% (1/168).

## Discussion

Although TORS has been touted to have advantages over alternative treatment modalities including decreasing morbidity associated with open procedures, reducing hospital admissions, and improving survival (1), there remains concerns over the potential short-term hemorrhage and mortality risk. However, the literature on this topic remains incomplete with only a few studies specifically assessing perioperative mortality (1, 13). In addition, to provide adequate counselling to patients electing to undergo TORS, clinicians should be able to provide accurate estimates of mortality risk. Our study is the first, and largest, study to analyze national hospital-based data to specifically assess mortality risk within the acute perioperative period. Our finding of a 30-day perioperative mortality of 0.6% is within the mid-to-lower range (0.07-3.3%) of the current literature **(** **Table 1** **)** (2, 7, 14, 16–19). To provide context, the 30-day mortality rate for neck dissection alone has been reported between 0.5-1.3% (22, 23). We chose to focus on 30-day perioperative mortality to adequately capture only those deaths within the acute perioperative period related to surgery alone. Presumably, adjuvant treatment would not have commenced within that period and therefore was not considered a confounder in our estimate. Although there was an expected increase in 90-day perioperative mortality to 0.9%, this was not vastly different from the 30-day mortality rate. Given that adjuvant treatment should have been initiated, or completed, within this time frame, the 90-day mortality rate provides an estimate of both delayed surgical events and those related to adjuvant treatment. The second largest study to provide an estimate of perioperative mortality using NCDB data from 2010-2015 demonstrated a 1.4% 90-day perioperative mortality risk (1). Although our rate was 0.5% lower than the reported rate in this study, we had included an additional year of data and used different inclusion/exclusion criteria. For example, unlike Nguyen et al. (2020), we excluded all patients that had TORS, but required conversion to an open procedure (1). It is possible that these patients may have had larger tumors and/or were more challenging to resect transorally due to issues related to access and therefore were at a higher risk of postoperative bleeding or airway compromise.

The only variable found to be associated with 30-day perioperative risk on UVA was age≥65. The role of upfront TORS in elderly patients with OPSCC remains controversial. It is apparent that over time the demographic profile of the typical patient with HPV related OPSCC is changing (24, 25). Recent modelling predicts that the incidence of HPV+ OPSCC in the USA will rise in patients 65 years of age and older from 40.7 to 71.2 per 100,000 by 2029 (25). Therefore, we decided to evaluate age≥65 as a cut-off for assessing perioperative mortality. With several trials demonstrating no survival benefit to using chemotherapy for treating head and neck cancer patients older than 70 years (26, 27), some authors have suggested upfront TORS may provide an effective treatment modality for this cohort (28). However, given that we found the risk of perioperative mortality is 3.4 times higher in those patients older than 65 years of age undergoing TORS for T1-T2 OPSCC, caution should be taken when selecting appropriate candidates for surgery. Our analysis was not able to assess cause of death, and as such, it may be that many of the patients in this category may have had non-bleed related deaths such as stroke or MI which are not infrequent in a cohort of patients that are treated with neck dissection (29). Nonetheless, the importance of pre- and perioperative measures to reduce the incidence of life threatening hemorrhage should be emphasized in this older cohort (i.e. timely discontinuation/restarting of anticoagulant medications, TAL, possible prophylactic tracheotomy, using alternative treatment modalities for tumors in close proximity to major vessels, etc) (8, 9). The higher risk of mortality within this age group should also be fully explained during pre-operative counselling as patients weigh the potential surgical and non-surgical treatment options available to them. The additional benefits of avoiding primary radiation or chemoradiotherapy may be attenuated in the older population given the excellent short-term safety profile of this primary treatment and limited long-term side effects in a more elderly cohort. Studies comparing mortality in the non-surgical group for chemoradiation or radiation only groups will help determine if surgical complications or non-surgical issues contributed to mortality. Although the exact cause underlying this association is unknown, one recently published study found a similar association due to younger patients presenting with more advanced nodal disease (30). In agreement with this study, we found that younger patients (<65 years) were observed to have higher N-category (N2/3) disease (<65 years: 1920/2563 vs ≥65 years: 643/2563; p<0.01) and therefore this association may be a reflection of the previously addressed impact of age on perioperative mortality.

These data must be interpreted in the context of the study design. First, as with any retrospective study, we are unable to account for variables that were not captured within the NCDB dataset. Second, our results are not generalizable to all patients undergoing TORS as we specifically examined only those patients with T1-T2 OPSCC that were selected for surgery. Lastly, given the nature of using a large database, the granular data regarding the specific causes of mortality are lost. Therefore, although most deaths within the acute perioperative period may be attributable to surgery, the specific cause of death was not captured. Likewise, we were unable to determine the surgical technique employed for each patient (i.e. TAL, sequence of neck dissection with respect to TORS resection of the primary tumor, prophylactic tracheotomy, etc) including trends with time or the experience of the TORS surgeon. Furthermore, although overall rates of postoperative hemorrhage are not significantly different with TAL, there is a trend towards lower rates of life-threatening hemorrhage (7). Amendment of ECOG3311 protocol to strongly recommend TAL occurred in January 2016 (2). Therefore, the rate of TAL within the NCDB dataset from 2010-2016 is unknown.

Contemporary treatment strategies for early-stage OPSCC continue to remain varied in approach. De-intensification of both primary and adjuvant treatment of early-stage HPV+ OPSCC through altering radiation dose, fractionation, types of concurrent chemotherapy, amongst other strategies is the primary aim of many recent and ongoing trials (2, 31, 32). The potential morbidity and mortality associated with each treatment should be weighed against changes in survival. With respect to acute adverse events, the overall morbidity of primary chemoradiation remains elevated in comparison to radiation alone (33). However, as evidenced by the recent NRG-HN002 trial, omission of concurrent chemotherapy in primary treatment of early-stage HPV+ OPSCC resulted in reduced progression free survival with similar impact on quality of life/swallow outcomes (33).

With respect to primary surgery, our study demonstrates that TORS remains a treatment option with low perioperative mortality rates and excellent overall survival. However, it should be highlighted that approximately two-thirds of our patient cohort required some form of adjuvant treatment despite low positive margin rates. This finding is consistent with other studies (1, 10, 15). This further underscores the need for rigorous patient selection to avoid operating on those patients that, based on preoperative imaging (i.e. radiologic evidence of extranodal extension), are more likely to require additional treatment. Although not a focus of our study, other authors have published on using a variety of patient specific and anatomic factors to select the ideal candidates for TORS (34, 35). For example, achieving adequate oropharyngeal exposure is critical to achieving negative margins and reducing the risk of intraoperative complications (34). As highlighted in our own study, caution must be also be exercised in the elderly population where perioperative mortality rates are elevated following TORS. Therefore, the optimal indications for TORS in treating OPSCC may be in younger patients (<65) with early-stage HPV+ OPSCC with adequate oropharyngeal exposure where dual and triple modality treatment can be limited or avoided.

## Conclusion

The use of TORS for primary surgical treatment of early-stage OPSCC carries an estimated 0.6% risk of 30-day perioperative mortality. This mortality risk remains relatively low but is 3.4-fold higher within an older cohort (≥65 years of age). Therefore, while the perioperative mortality risk of TORS for treatment of early-stage OPSCC remains low overall, we must remain judicious in selecting the appropriate candidates for surgery and be transparent about the inherent risks when counselling patients. Future studies should include specific cause related mortality, compare non-surgical treatment and include HPV status and tobacco use when feasible.

## Data Availability Statement

The datasets presented in this study can be found in online repositories. The names of the repository/repositories and accession number(s) can be found below: National Cancer Database.

## Ethics Statement

Ethical review and approval was not required for the study on human participants in accordance with the local legislation and institutional requirements. Written informed consent for participation was not required for this study in accordance with the national legislation and the institutional requirements.

## Author Contributions

Concept and design: JD, EG, and AE. Acquisition, analysis, or interpretation of data: JD, EG, and AE. Drafting of Manuscript: JD, ZH, TD, EG, and AE. Critical revision of the manuscript for important intellectual content: JD, ZH, TD, EG, and AE. All authors contributed to the article and approved the submitted version.

## Conflict of Interest

The authors declare that the research was conducted in the absence of any commercial or financial relationships that could be construed as a potential conflict of interest.

## Publisher’s Note

All claims expressed in this article are solely those of the authors and do not necessarily represent those of their affiliated organizations, or those of the publisher, the editors and the reviewers. Any product that may be evaluated in this article, or claim that may be made by its manufacturer, is not guaranteed or endorsed by the publisher.
